# An Optimum Principle Predicts the Distribution of Axon Diameters in Normal White Matter

**DOI:** 10.1371/journal.pone.0054095

**Published:** 2013-01-28

**Authors:** Sinisa Pajevic, Peter J. Basser

**Affiliations:** 1 Mathematical and Statistical Computing Laboratory, Division of Computational Bioscience, Center for Information Technology, National Institutes of Health, Bethesda, Maryland, United States of America; 2 Section on Tissue Biophysics and Biomimetics, Program on Pediatric Imaging and Tissue Sciences, Eunice Kennedy Shriver National Institute of Child Health and Human Development, National Institutes of Health, Bethesda, Maryland, United States of America; University of Alberta, Canada

## Abstract

Many important functional properties affecting nerve conduction are influenced by axon diameter. It is also known that the axon diameter distribution (ADD) in normal nerve fascicles is heterogeneous and skewed. A recent attempt to model and explain the parametric form of these distributions was based on biomechanical principles. Here we explore a neurophysiologically-based hypothesis that the observed ADD can be obtained by optimizing the information flow through a fascicle subject to reasonable anatomical and metabolic constraints. Specifically, we use a variational framework to find an optimal distribution based on the fascicle's channel capacity and informative upper bound (IUB), subject to constraints of fixed available fascicle cross-sectional area and fixed number of axons, to derive two novel probability density functions, which we then compare to other previously used distributions. We show, using experimental histological data, that the distributions based on this optimum principle outperform other distributions. Moreover, the new distribution that optimizes the IUB is extremely robust in fitting ADD data obtained histologically, making it well-suited for use in MRI techniques to measure ADDs *in vivo*, e.g., AxCaliber MRI.

## Introduction

Throughout the evolution of the central nervous system the benefits of increased brain size were offset by the cost of increased processing delays caused by signals having to travel longer distances. To compensate for this, different strategies evolved, most notably, myelination of axons and saltatory conduction, which greatly increase conduction velocity [2]. It is well-known that the speed of conduction along a myelinated axon scales with axon diameter [3], [4], so regulating axon diameter presents another way to overcome long-range conduction delays. However, within the brain the degree of myelination is observed to vary significantly within and among different nerve fascicles [5], and the axon diameter distribution (ADD) within fascicles can be heterogeneous, broad, and skewed. This implies that faster is not always better and that a more nuanced organizing principle might be at work. As the size of a given individual axon and its degree of myelination can change during development, and also adaptively in response to the level of activity of a given axon [6], [7], it is possible for a given species, through evolution, or an individual organism through adaptation, to tune and control the ADD to enhance nervous system performance and consequently survival.

It has long been established [3], [4], [8], [9] that a number of important functional properties affecting nerve conduction depend on the degree of myelination of an axon and its diameter. A number of subsequent studies have used physical principles to arrive at scaling laws of axons to describe these observations [10]–[12] (which should not be confused with the scaling laws describing relative volumes of white and gray matter [13], [14]). In Rushton [10] the focus was on deriving scaling relationships governing how different morphological features compare with axon diameter by assuming that all myelinated axons are dynamically similar (i.e., using the “principle of corresponding states”). In Basser [12] the same scaling relationships were obtained by maximizing the effective space constant of a myelinated axon while minimizing its effective time constant. In Wang et al. [11] comparisons were made between axonal properties across many different mammalian species.

The main goal of this paper is to provide a novel explanation for the skewed form of the existing ADD data using an optimum principle. Besides our earlier work [15] we are aware of only two similar attempts. In a recent work by Perge et al [16], the authors suggest the relationship between the axon diameter and the information rate by studying several categories of the fiber tracts with different functional and anatomical properties, and arguing that to maximize the information transfer one needs a broad distribution of fiber diameters, containing both the small and large ones. However, no parametric form for the ADD is derived. In an earlier work by Gov [17], biomechanical principles are used to derive a novel parametric form of the observed ADDs. More precisely, it is argued there that the observed skewed ADD is the result of the interplay between curvature bending energy of the membrane surrounding the axon core and active processes that remodel the microtubules and neurofilaments inside the axon. Here, we postulate that the observed skewed ADDs optimize information transmission or channel capacity, given the overall constraints of a fixed number of fibers and a fixed available area/volume. We use the calculus of variation to obtain the parametric form of this optimal ADD. While the main goal here is to help explain interesting neuroanatomical findings, we also emphasize that having a proper parametric form for ADDs has practical value, particularly in a newly developed MRI method for measuring ADDs *in vivo*, AxCaliber MRI [1]. In our previous implementation of AxCaliber MRI the gamma distribution is used as the parametric form for ADDs. For this reason we compare this new “optimal” model to the gamma distribution, as well as to other distributions proffered to find the most robust and accurate one for fitting ADD data.

### ADD Modeling Assumptions and Considerations

Two assumptions are inherent in our approach. The first is that the degree and quality of myelination is similar for each axon within a given cross-section, so that the axon diameter is the main determinant of the properties of signal propagation along such axons. The second is that the cross-sections of all axons are geometrically similar so that the *g-ratio* or the ratio of the inner to outer diameters of myelinated axons is a constant (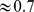), which is supported by experimental evidence [4], [10]. In these studies there is no clear trend in terms of the dependence or the g-ratio on the diameter, however, the values within the same nerve or the fascicle can still vary significantly. Hence, in the rest of the manuscript we assume that only one parameter, 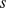, characterizes an axon's radial size. Additionally, there are four relationships between axon diameter and its functional properties that we consider when deriving the form of the ADD: (1) the larger the axon's inner diameter, the shorter the duration of the action potential and refractory period, and hence the larger the maximal frequency of firing [18]. Here we assume that a simple power-law function adequately describes this relationship, motivated by the fact that the refractory period, (i.e., the inverse maximal frequency), is related to the speed of propagation [18]; (2) the larger the axon's inner diameter the higher the conduction velocity, 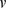. This relationship can be summarized as 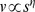, where the reported values for 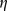 range between 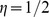 (usually for unmyelinated axons) and 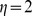, with 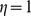 often reported for myelinated axons [3], [8], [9]. The values of 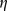 do not only depend on myelination, but also on the location of the fascicles and the species under study; (3) the smaller the outer diameter of the axon the more axons can be packed together per unit area. This is self-evident, but it could also be argued that packing is not merely dependent on axon diameter but on the ADD. Clearly, a pack of cylinders of given fixed diameter is not as space filling as a pack of cylinders with a more heterogeneous ADD; (4) the smaller the axon's inner diameter the lower the metabolic energy required to maintain it. Assuming the mitochondrial density is uniform in the axoplasm, the metabolic energy needed to sustain the axons within a fascicle should be roughly proportional to the total axonal volume, hence the energy per unit length of an axon will be proportional to its inner cross-sectional area.

So, according to considerations (1) and (2) above, axon diameters should be as large as possible to maximize information transfer. However, both (3) and (4) suggest that there are benefits to making them smaller. The mathematical framework presented below allows us to balance these multiple objectives.

### “Optimal” Axon Diameter Distribution

Different optimization strategies have been employed to justify axonal sizes and network connectivity. In Chklovskii et al. [19] this has been done at the level of branching points of a single axon, and an expression is derived for the optimal axon diameter, which minimizes the combined cost of conduction delay and arbor volume (similar to points (2) and (3) mentioned in the Introduction). In Chen et al. [20] it was shown that the connectivity in the Caenorhabditis elegans (*C. elegans*) is organized in such way as to minimize the wiring costs. In our case, we study the optimization of information transfer at the level of a fascicle with respect to the ADD [15].

#### Information transmission capacity of axons and fascicles

Early attempts to apply information theory to nerve transmission were characterized by discrepancies in the assumed information rate [21], ranging at the low end from 0.3 to 5 bits per second per cell [22], [23] to 4000 bits per second per cell at the high end [24]. One reason for these discrepancies is that making different assumptions about how the brain codes information leads to different estimates of the rate of information transferred. In order to rigorously apply information theory to neurotransmission one would need to address the question of coding, specifically how the brain represents given symbols to be transmitted. Two of the most common neural coding schemes are (1) temporal, or time coding, and (2) frequency, or rate coding. Under the assumption of temporal coding [25], [26] as well as constant, lossless and dispersionless action potential propagation (no jitter), the conduction speed has no influence on the transmission of information since the spike departure/arrival times are preserved. Also, without jitter axons act like noiseless channels. McCulloch and MacKay [27] have calculated the channel capacity of an axon assuming both types of coding, the pulse (rate) code modulation and pulse interval (temporal) modulation. For a binary modulation system the limiting capacity is simply the maximal attainable pulse rate, and for pulse interval modulation, they derive a formula for the channel capacity, 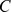,
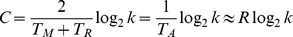
(1)where 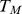 and 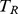 represent minimal and maximal permissible values of the time interval, 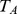 is their arithmetic average, 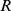 is the average firing rate, and 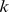 is the total number of discrete temporal intervals (states). Thus, in both of these cases channel capacity is roughly proportional to the firing rate, 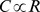.

Another measure that can characterize the information capacity transmitted by axons is the informative upper bound (IUB). Here we use the form presented in Zador [28]. This upper bound in a discretized spike train is obtained when there is no noise, spikes are independent, and the spike rate is low compared to the inverse bin size, 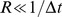, i.e., when spikes are Poisson distributed. Then, the probability of observing a spike, 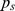, in a given bin is proportional to the firing rate, 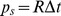. Under the Poisson process assumptions that the spike observations in successive bins are independent and 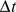 is small, 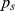 is small and the contribution to the entropy of not observing a spike, 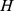, is negligible. Then, this entropy is simply 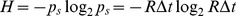, hence, the information rate (information per 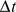) is,
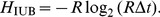
(2)


#### Variational approach to derive “Optimal” ADD

To find the ADDs that optimize the informative upper bound and the channel capacity subject to given neurophysiological constraints we use variational calculus [29]. As mentioned above, we use the experimental fact that the g-ratio does not show a strong dependence on axon size and is relatively constant (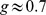) [4]. Together with an assumption that mitochondrial density is uniform in the axoplasm, it enables us to lump together the volumetric and metabolic constraints, since the metabolic energy per unit length needed to sustain the axons within a fascicle will be proportional to the total axonal area, 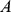. The variable being optimized in the variational framework is the ADD itself, a function that we designate as 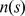, representing the number of fibers whose diameter lies within the interval 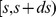. We optimize the information transmission along a fascicle whose ADD is given by 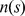 using the constraints of a fixed total number of fibers, 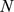, and/or a fixed total cross-sectional area of the fascicle, 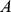. Mathematically, we seek 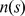 that optimizes the functional, 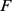,
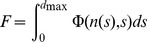
(3)with an arbitrary upper limit of integration, 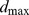, assumed to be larger than the largest possible axon diameter. 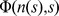 can be written as
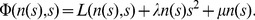
(4)which incorporates the “Lagrangian”, 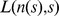 (which, in a more general case can be a function of 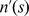) and the two constraints
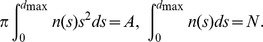
Thus, the Lagrange multipliers 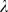 and 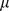 can be considered as the weighting factors of the constraints of fixed total cross sectional area and fixed total number of fibers, respectively.

We first optimize 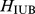, given in Eq.(2), hence, 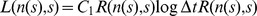, where 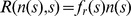 is now the total rate observed in the fascicle, 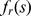 is the maximal firing rate for an axon with size 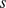, and 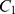 is an arbitrary constant, which can be ignored. Despite its functional significance, there is still no agreement on what the appropriate form for 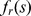 should be. When we model it by a simple power-law, 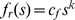, we arrive at a compact, closed-form solution for the optimal ADD,
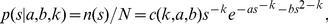
(5)where 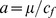, and 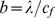 are the model parameters, and 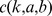 is the normalization constant for which we can find a closed form expression only for special cases. For example, relaxing the constraint that the fascicle area be fixed (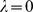), and for 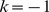, 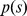 assumes a very simple form,
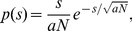
(6)a special case of which is the gamma distribution.

For an arbitrary real value of 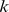 and arbitrary moments, an analytical expression for 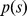 could not be found. However, for 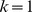, we obtained analytical expressions for 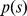 and all of the moments. We use this important special case in our model comparison, to which we refer as the informative upper bound distribution (IUBD); we describe its statistical properties in greater detail in the next section. For some other values of 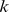 one can also obtain analytical but more complex expressions. For example, for 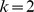 it is possible to obtain a simple expression for the normalization constant (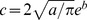) but not for the moments.

As an additional example we also optimize the Lagrangian which is based on channel capacity, i.e., 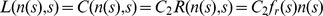, which essentially is the total rate that a fascicle can handle. In this case it is necessary to modify the constraints since each term in Eq.(4) contains a common factor of 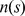, which can thus be factored out and cannot be determined variationally. For this reason we relax the constraint of having a fixed number of axons in each fascicle by introducing a third constant, 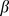, whose value should not be too far from 1, and which for 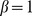 re-imposes the original constraints. Then, 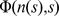 can be written as
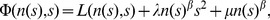
(7)and the functional form that optimizes this new Lagrangian and constraints is
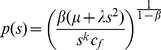
(8)which can be written in simpler form as
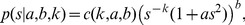
(9)which is the form we use in our model comparison. Besides 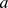 and 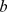, we also use the normalization prefactor, 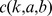, as a third fitting parameter, and since it essentially optimizes the total rate, we refer to it as the total rate distribution (TRD), which in the rest of the manuscript will refer to the special case 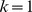.

More elaborate optimization frameworks could include other constraints, as well as other forms of the “Lagrangian”. While the distributions obtained in (5) and (9) should not be viewed as definitive, they represent a rational framework to achieve a compromise between competing biological objectives, providing a roadmap for including other *a priori* information, physical constraints, and new knowledge about neural coding strategies.

### Statistical Properties of IUBD

Properties of 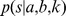 in Eq. 5 can be expressed analytically for 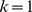 and in that case the moments, 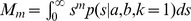, are
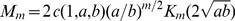
(10)where 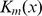 is the modified Bessel function of the second kind (Basset functions). The modified Bessel functions of the second kind can be expressed in terms of the modified Bessel functions of the first kind as
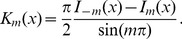
(11)The normalization constant is obtained for 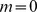, using 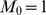, i.e.,
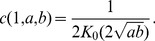
(12)Using Eqs. 10 and 12 the two-parameter form of the IUBD distribution is derived,
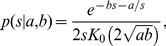
(13)yielding also a two-parameter formula for the 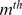 moment of the distribution, which can now be written as
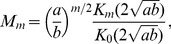
(14)the characteristic function for IUBD,
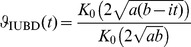
(15)and the moment generating function, 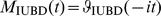, i.e.,
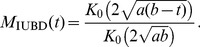
(16)


### Models Used for Comparison

Below, we fitted five different parametric models to previously published histological ADD data. Besides the distributions proposed here, we use three other plausible probability distributions, the gamma distribution (GD), log-normal distribution (LND), and a distribution based on a physical model (PMD) [17]. Below we give a short summary of each model. The remaining two models are the ones described above (IUBD and TRD), the parametric forms of which are given in Eqs. 5 and 9, respectively. Since in their full form they have three fitting parameters, we fix 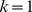, so that the final comparison is made between the models with an equal number of fitting parameters. All candidate distributions are essentially two-parameter models, but since normalization constants are not directly available for some of the models, the normalization parameter, 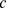, is added to each of them.

#### Gamma Distribution

The gamma distribution is widely used and found in many problems involving waiting times due to its relation to the Erlang distribution. We use the standard form written as
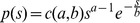
(17)where 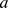 represents the shape and 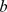 the scale parameter, and the normalization constant is equal to 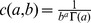. We include the gamma distribution here because it is currently used in our implementation of AxCaliber MRI.

#### Log-normal Distribution

The log-normal distribution is ubiquitous [30], [31]. It has been argued that many distributions arising in nature are better fit by the log-normal rather than the normal distribution [32] mainly because the product of random variables leads to this distribution, according to the central-limit theorem. For example, the log-normal is used, for instance, in granular media, where large pieces can crumble into smaller ones, each roughly a fixed fraction of the original. This distribution is not limited to materials and biology; another well-known example is the Black-Scholes model in which stock price fluctuations are log-normally distributed (although the actual data deviates significantly from the model). The mathematical form that we use here is
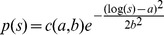
(18)where 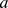 represents the location and 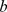 the scale parameter, analogous to the mean and variance, and the normalization constant is 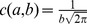.

#### Physical Model Distribution

In Gov [17], a physical model is proposed which essentially uses biomechanical reasoning to explain the observed ADDs. It posits that the actual distribution is the result of balancing the curvature/bending energy of the membrane surrounding the axon core and active processes that remodel the microtubules and neurofilaments inside the axon. The parametric form of this distribution can be written as,
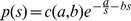
(19)where the parameters 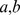, and 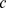 can be expressed in terms of various physical parameters as described in Gov [17].

## Materials and Methods

### ADD Histological Data

**Figure 1:**
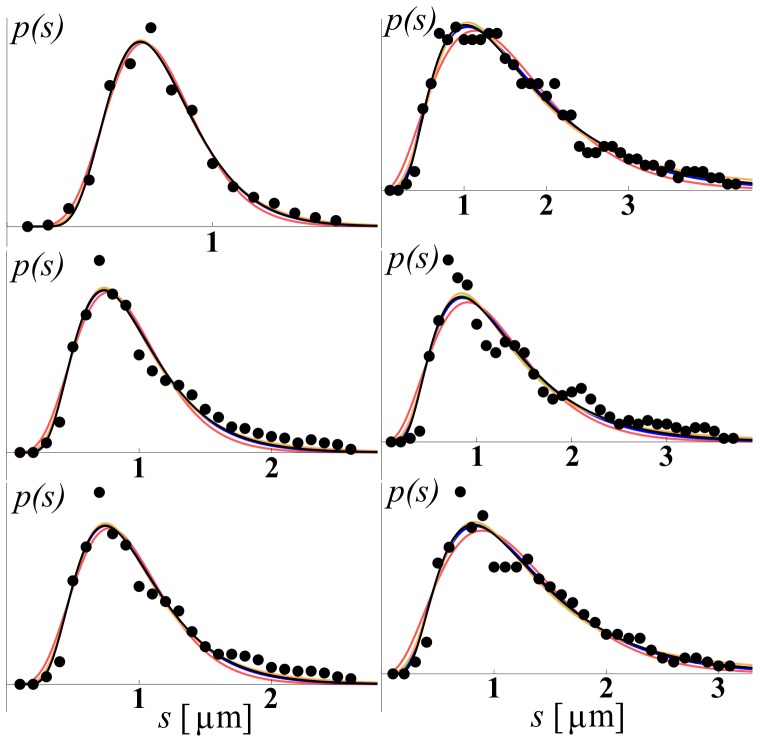
Examples of the model fits obtained using EMD1 data [1] (solid circles). The fitted models are displayed as follows: IUBD (black), TRD (orange), GD (red), LND (green), PMD (blue). The x-axis designates the size of the axon in microns (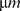); the y-axis is in arbitrary units. The complete set of fits is provided in the supplementary material, together with the anatomical information about each individual data set.

**Figure 2:**
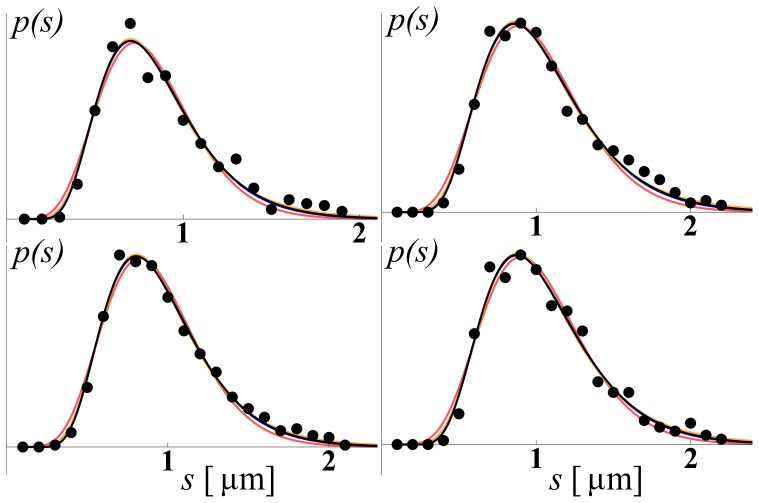
Examples of fits obtained using data EMD2. The legend is the same as that in figure 1.

**Figure 3:**
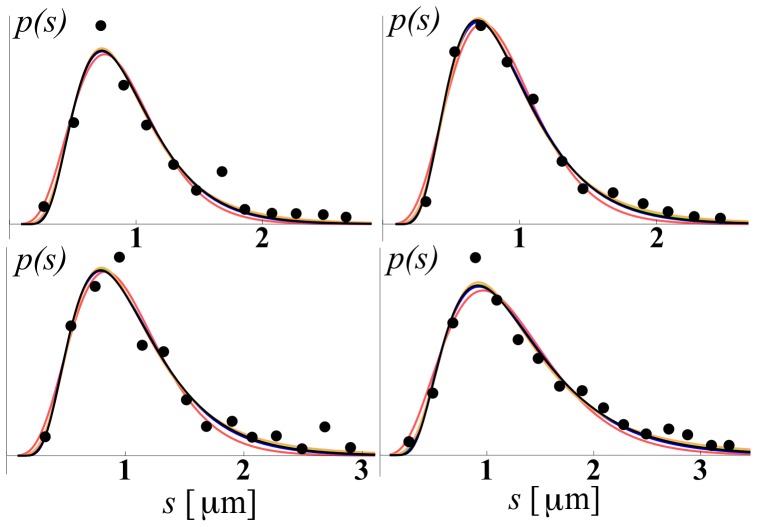
Examples of fits obtained using data EMD3. The legend is the same as that in figure 1.

We use three different experimental data sets, each containing several axon size histograms, or ADD curves, obtained by analyzing the electron micrograph (EM) cross-sections obtained from different regions of the brain. We label these data sets simply as EMD1, EMD2, and EMD3. The first data set, EMD1, contains the ADD curves previously described in Ong et al. [33]. They were obtained from seven mouse brain regions as follows: a) dorsal corticospinal (dCST), b) gracilis (FG), c) cuneatus (FC), d) rubrospinal (RST), e) spinothalamic (STT), f) reticulospinal (ReST), and g) vestibulospinal (VST). Each of the regions had 5 replicates, hence, in all, there were 35 ADDs. The second data set, EMD2, contains six ADDs obtained from different but consecutive regions of excised and fixed rat corpus callosum obtained after the samples were scanned *in vivo* using the AxCaliber MRI method [1]. Data correspond to six consecutive sections of the corpus callosum, going from anterior to posterior, i.e., from genu to splenium. The third data set, EMD3, published in Aboitiz et al. [34], is obtained from a human corpus callosum, of an individual who died from non-neurological disease and which was acquired within 12 hours after the death. The five ADDs represent histograms of fiber diameter frequencies in the following regions: a) genu, b) anterior body, c) midbody, d) posterior body, and e) splenium. “Anterior body” represents a region including the posterior genu and the anterior midbody, and “posterior body” represents a region including the posterior midbody and the isthmus. A sample of these data sets, together with the model fits, is shown in Figures 1, 2, 3, while the complete data sets and fits obtained on all 46 ADDs are given in the Figures S1, S2, S3. The values of the fitted parameters are reported in Table S1.

### Model Fitting and Comparison

We use a two-step process to fit any model to the ADD data. In the first step we select the best dynamic range for each of the data sets. This was necessary since many of the data sets contained points that extend well beyond the range at which the ADD curves have already decayed close to zero. Our selection rule in removing the large diameter tails was to find the first data point in which the histogram frequency has decayed below the 1/20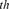 of the peak value, including it and the following two points, and excluded the remaining large diameter data. The same is done using the rule of 1/50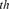 of the maximum and taking the next 5 points, hence including more of the outliers (data not shown). We obtained virtually the same results in both cases and only the former data are included in this manuscript. In the second step, we perform an extensive search for the best initial guesses of parameters, starting with a wide domain of randomly chosen values, but then narrowing this random search based on the least square error (LSE) obtained in each trial. The main reason for this extensive search is that several of the distributions fail to fit the data properly when arbitrary initial values (guesses) are used. The fits were performed using non-linear regression in Mathematica® 8.0 (Wolfram Research) with functions NonlinearModelFit and FindFit, whose performance on difficult non-linear regression tasks is usually deemed better than most other statistical packages (e.g., see [35], [36] for comparisons that utilize NIST benchmark data sets). In the end, after an extensive search, we were able to obtain reasonably successful fits for each of 46 ADDs and for each of the five models (see Table S1 in the Supporting Information).

After the fitting stage we compare the models using the goodness of fit estimates where our primary goodness of fit measure is the total LSE expressed as the percent of the total energy in the data (sum of squares of data values), 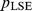. In essence, this energy is equal to the LSE when the model is just a flat line, 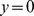. When 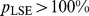, we declare such a fit a failure, which occurs frequently in some of the models if the initial conditions are not carefully chosen. We also use additional goodness of fit measures of the quality of the fits, such as the correlation coefficient, 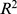. This measure can be quite misleading when using non-linear regression; however, all our models fit the data well enough that the correlation between the model and the data is a reasonable measure of the quality of these fits. We also report Akaike and Bayesian information criteria (AIC and BIC, respectively), noting that the lower their value the better the fit. They are mainly useful when comparing models with differing numbers of free parameters, hence, here they are not as important, since all the models that we study have the same number of parameters and can be very effectively compared using LSE. AIC and BIC are discussed here because they could become more useful when a larger family of candidate distributions is considered.

To compare different models more precisely we perform a large number of additional non-linear fits using two types of randomization methods: (a) the jackknife (JK) resampling scheme that enables us to make a statistical comparison for each individual ADD, and (b) initial parameter value randomizations, or simply randomized initializations (RI), which addresses the sensitivity of some models to the initial values of the parameters.

The jackknife is a resampling scheme that we deemed best suited, albeit not ideal, for our ADDs to provide non-parametric error estimates and statistical comparisons between our goodness of fit measures. While it appears that the wild bootstrap and parametric bootstrap might provide a better way to produce randomized replicates of ADDs, these methods already presume the parametric form of the model and thus would not be adequate for our model comparison purposes. Our implementation of the jackknife randomization differs in two ways from the standard method, where a single data point is chosen randomly and removed from the original sample. First, we explore situations in which more than one point is removed, second, we remove points from our ADDs, not the actual sample, and then use the non-linear regression to obtain a new jackknife sample. We refer to our JK randomizations as JK1, JK2, etc, where the number indicates how many points were randomly removed from the ADDs prior to regression. We repeat the jackknife procedure 100 times, noting that for JK1 there will be a high likelihood that multiple identical samples will appear (since the ADDs have only about 20–40 points). The initial parameter values for each JK fit were those that produced the best fit in the extensive search we performed in the prior stage. This procedure allows us to estimate errors and statistically compare all of the goodness of fit measures used, instead of relying on a single value attained in the original fits. With the jackknife we can then perform statistical model comparisons on each individual ADD, instead of just relying on the statistical comparisons using all 46 ADDs. This capability is important since our data set combines ADDs from three different mammalian species and acquired by three different laboratories, hence the variability in our sample is expected to be much larger than it would be in a carefully controlled ADD measurements acquired specifically for model comparison. In our statistical tests we have usually compared two samples of 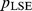, one from IUBD and one from the other distributions, and used a one-sided t-test with alternative hypothesis that the 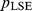 of IUBD is lower than the 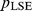 of the other distributions. The significance level we use is 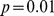.

Knowing that it was difficult to obtain reasonable fits for some of the distributions we were comparing, and that in many situations one cannot afford to perform the extensive search we did, we explored the fitting robustness of these models. This robustness is an important issue if these models are to be used in any practical applications, e.g., AxCaliber MRI, in which a distribution of choice is expected to fit the data reliably in a large number of voxels in different anatomical regions without a need to fine tune the initial parameter values. For this reason, we use the RI, in which the initial parameter values are randomized around the optimal ones, 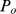, found after an extensive search to produce a satisfactory fit. We generate the randomized values, 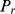, using the formula
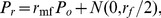
(20)where 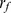 is the randomization factor indicating how much of a spread around the optimal initial value, 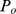, one should have, and 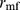 is a multiplicative factor chosen randomly (with uniform probability) between 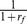 and 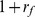. This randomization is repeated 1000 times. For each replicate, i.e., a choice of the initial parameter values, a non-linear fit is performed and its quality is quantified in terms of several parameters. In RI many of the fits will fail, in many cases with nearly infinite LSE differences between the model and the data. In this work, any fit for which 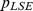 is equal to or greater than 100% is deemed a “failed” fit. Using RI we can then estimate the percentage of failed fits obtained for each of the models and each of the data sets.

## Results

As mentioned, for each of the five models described above we were able to obtain a relatively good fit after an extensive search; some of the fits are shown in Figures 1, 2, 3. Due to space limitations we show only a representative sample for each of the three different EM data sets, EMD1-3; however, the full set, with fits for all 46 experimental EM ADD curves, is provided in the supplemental figures S1, S2, S3. From these figures we see that, considering that EM ADD data are quite noisy, all five models fit the data reasonably well and hence it is not easy to distinguish between different models by visual inspection alone. However, even visual inspection indicates that the gamma distribution (red lines) does not fit well, particularly for small diameter fibers. To assess each of these models quantitatively, we perform detailed goodness of fit and statistical comparisons among them.

### Model Comparison

**Figure 4:**
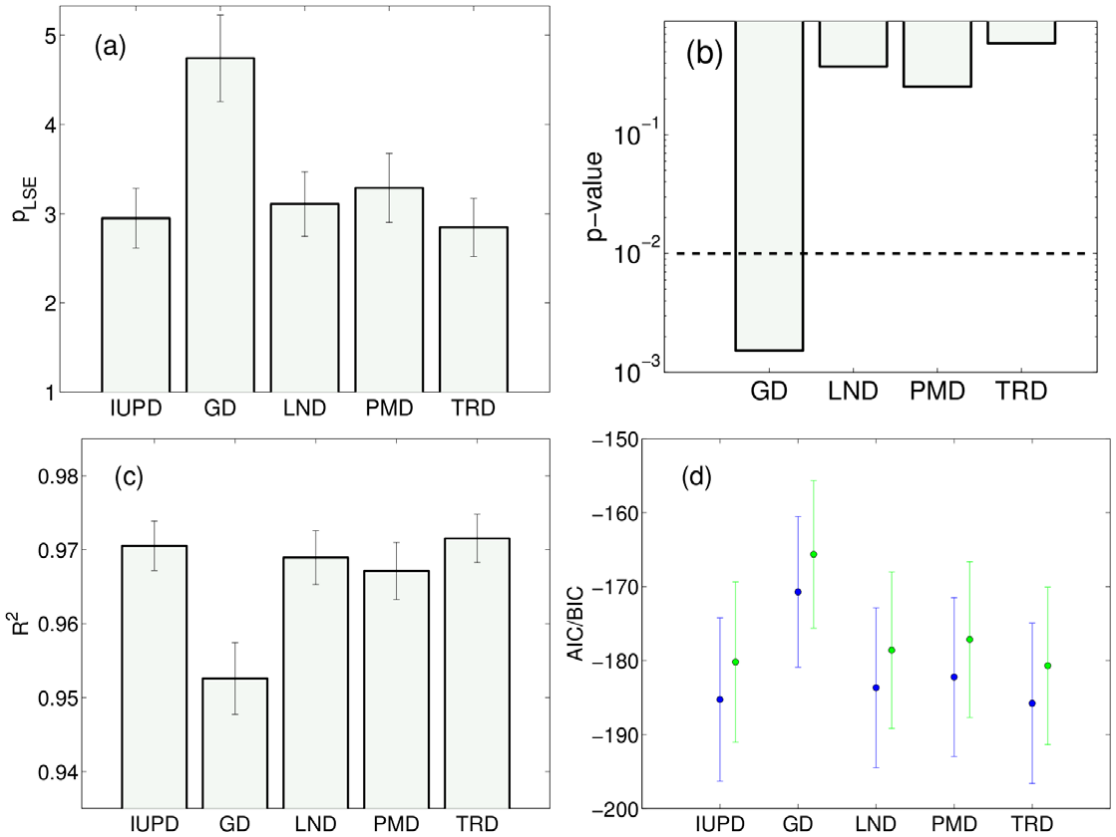
Model comparison using (a) 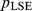
 **with reported mean and SE across all 46 data sets; (b) p-values obtained using one-sided paired t-test between IUBD and other distributions.** At significance level p = 0.01 (dashed line) only GD can be declared to perform worse. Similarly we show the mean and SE across 46 data sets for (c) 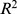 measure, and (d) Akaike (blue) and Bayesian (green) information criteria.

Direct comparison between the models in terms of 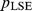 show that the two distributions derived here, IUBD and TD, perform best. The mean and the standard deviation of 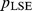 across 46 different data sets were, in increasing order: TD: 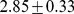, IUBD: 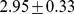, LN: 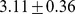, PMD: 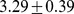, and GD: 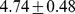; these values are displayed in Figure 4a. Even though TD outperforms slightly the IUBD (but not by a statistically significant difference), at the start we determine that the IUBD will be our distribution of choice to be eventually used in practical situations, based both on the quality of the fits and its robustness, as will be shown below. We thus focus on making statistical comparisons between IUBD and other distributions, including TD. When making a paired t-test comparison between IUBD and the remaining four distributions we obtained the following p-values: p = 0.0016 for GD, p = 0.37 for LN, p = 0.25 for PMD, and p = 0.59 for TD (Figure 4b). Hence, only the difference with GD would be declared significant for a reasonable significance level, and which can partly be attributed to low testing power when dealing with only 46 data points. Figures 4c and 4d, display 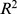, AIC and BIC quality of fit measures, but provide essentially the same ranking among the models as 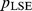.

**Figure 5:**
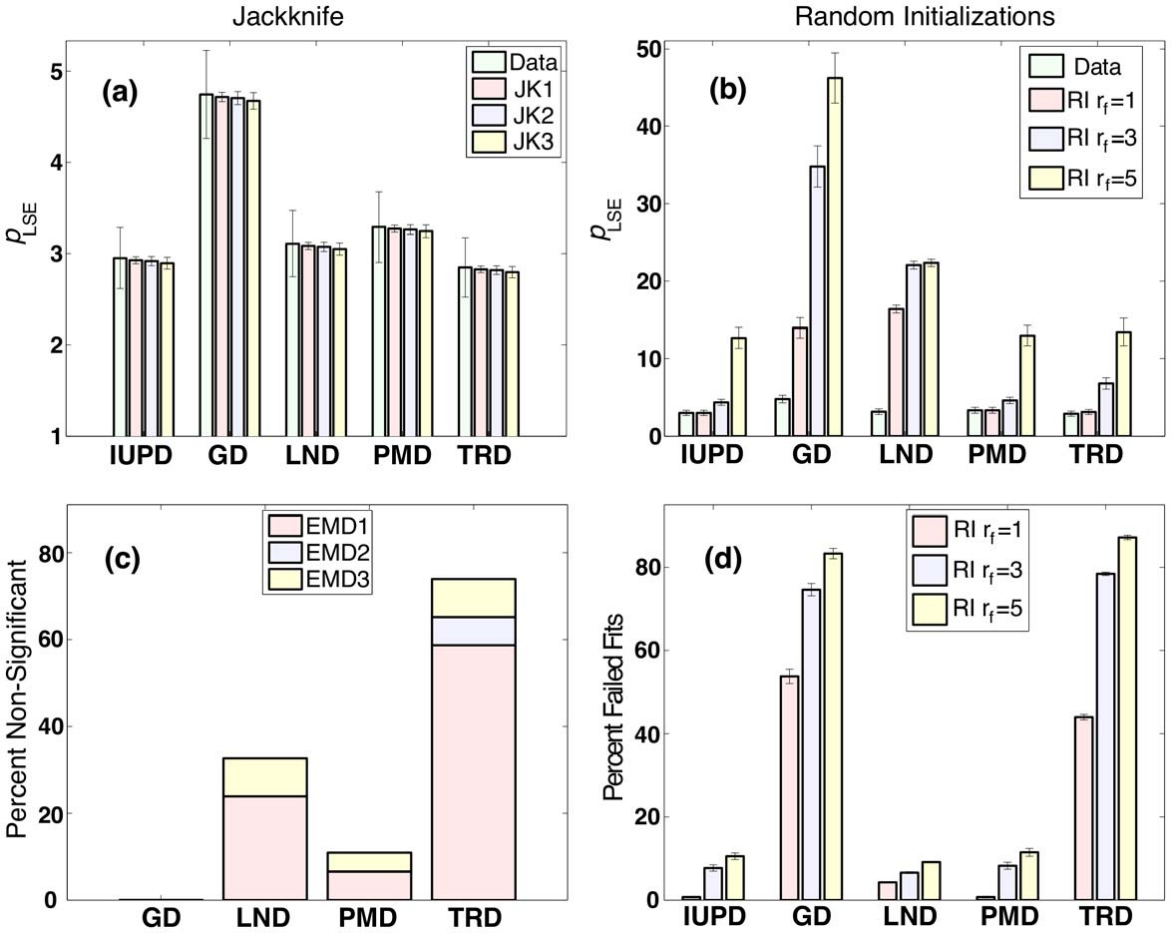
The left column shows the results obtained using jackknife, and the right column shows the results obtained using randomized initial conditions with the randomization factor 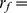
 **1, 3, and 5.** In (a) the mean values of 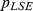 across 46 ADDs from figure 4 (labeled “Data”) are compared to the values obtained from 100 jackknife randomizations with varying numbers of points removed (JK1, JK2, and JK3). Error bars indicate SE for the original data, while for JK they are the estimates of such error. (b) Mean 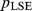 obtained from the “successful” fits among all RI replicates, and across all data sets, with SE reported across 46 data sets. (c) Percentage of ADDs, among 46, in which the IUBD 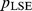 failed to be declared significantly lower. Each bar is colored to indicate how the ADDs are divided among different data sets. (d) Percent of failed fits among 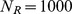 RI replicates with mean and SE reported across the 46 ADDs. N.B. differences in y axis scaling in (a) and (b).

To provide a further comparison we use randomization strategies, with most of the results summarized in Figure 5, so that the left column shows the results obtained using jackknife, and the right column shows the results obtained using randomized initial conditions with the randomization factor 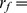
1, 3, and 5. In Figure 5a we show the mean least square error (LSE) percent differences between the data and the model, averaged across 46 ADDs as in figure 4a (labeled as “Data”), but now compared to the values obtained from 100 jackknife randomizations with varying numbers of points removed in each replicate (JK1, JK2, and JK3). Error bars indicate the standard error (SE) for the original data, while for JK they are the estimates of SE. Note that the JK estimates of the SE are much smaller than the SE of the original data, partly due to variability originating from physiological, anatomical, and methodological differences among the various ADD groups. However, there is also an inherent bias of our JK to underestimate the errors. Similarly, in figure 5b we compare the original LSE errors to those obtained from the “successful” fits among all RI replicates. The mean 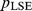 obtained, as expected, increases dramatically with the increased range of randomized initializations. Note that the increase in 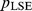 does not come from the presence of “failed” fits, i.e., the maximal allowed 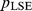 in the reported sample is less than 100%. Hence, the performance of GD and TD appears better than it really is since these two distributions had the largest percentage of the failed fits (see figure 5d), and the data shown here is only the average over approximately 20% of the lowest LSE.

When using a statistical comparison between 100 JK replicates of the mean 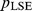 (hence, in total 100
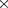
46 JK replicates are required), the 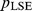 for IUBD was always (for JK1, JK2 and JK3) declared statistically better than GD, LND, and PMD, but never better than TD. This, however, can be due to the fact that in our implementation of JK the true variability in data is underestimated. A more useful approach would be to perform JK on individual ADDs, creating 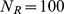 replicates for each curve. When making the statistical comparisons between JK replicates for each ADD we identified the data sets in which IUBD failed to be declared significantly lower. In figure 5c we report these as a percentage of all 46 ADDs. Each bar is colored to indicate how such ADDs are divided among different data sets, showing that IUBD was declared better than GD for all data sets, while for EMD2 it also was better than LND for all ADDs. For EMD1 and EMD3 a portion of ADDs failed to declare IUBD better than LND or PMD. More specifically, IUBD failed to be better in 16 ADDs (12 ADDs in EMD1 and 4 in EMD3) than LND, and in 5 (3 in EMD1 and 2 in EMD3) than PMD. In particular, these failed cases seem to be focused on dorsal corticospinal (dCST) in EMD1, where the fiber sizes were on average much smaller than in other regions, and on the most anterior and posterior regions of corpus callosum in EMD3. Further information on the individual ADDs in which IUBD failed to be declared more significant is provided in the supplemental material. Note also that in the majority of the cases and in all three data sets, IUBD failed to be declared better than the TD; however, we put less emphasis on the differences between the two, since both were derived in the same framework here. Note also that not-having IUBD declared better does not mean that the other distribution would be declared better as the more likely declaration would be that of null-hypothesis holding true, i.e., the distributions not having significantly different performances. Finally, in figure 5d we report the percent of failed fits among 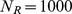 RI replicates with mean and SE reported across the 46 ADDs which show that the GD and TRD are highly unstable, while the IUPD and PMD had very comparable robustness. These findings can change if a different fitting procedure is employed.

## Discussion

The main goal of this paper is to introduce a novel concept to explain the observed skewed ADDs. Apart from arguments that such distribution should be log-normal, as in many distributions occurring in nature and granular media, or the structural physical constraints as in PMD, we argue here that a good neurophysiological reason for their shape is that it facilitates optimized information transfer and capacity along bundles of axons. We used methods of variational calculus to find the optimal ADD, 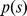, for which the total fascicle's informative upper bound or channel capacity is maximized given the constraints of fixed area and the number of fibers (or having 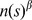 fixed). The constraint of having a fixed area encompasses not only the volumetric constraint but also a constraint of fixed metabolic energy per unit length of an axon. Note also that even though our implementation uses a formula for an area of a circle, our volumetric constraint would also apply to other 2D shapes, as long as the variability in sizes is not strictly limited to only one of the two dimensions. Our approach does carry an assumption that the packing density is not dependent on the size. For rigid shapes, packing issues can significantly alter the needed volume/area, however, from the known EM data, it does appear that the axons are efficiently and uniformly packed within a given cross-sectional area.

Since we did not have a large pool of EM ADDs available, we were forced to concatenate data from different groups, using white matter of different mammals (mouse, rat, human) and from different anatomical regions in each. Such variety is expected to increase the variability in data, thus reducing our ability to make a fine distinction among different models. As indicated by the results of individual ADD comparisons (see figure 5c), it appears that ADDs in nerve fascicles in different anatomical positions can be guided and organized by different principles. Studying this hypothesis further would require acquisition of many EM replicates from the same anatomical position and the same type of brain, and performing the same model comparison presented here.

As can be gleaned in some of the ADD curves presented in Figures 1, 2, 3, and in particular, if one looks at a complete ADD data set provided in the supplementary material in figures S1, S2, S3, some of the ADDs appear to have multiple modes. For example, the ADDs measured in gracilis (in Panel 1 of Figure S1) and vestibulospinal tract (VST) (in Panel 2 of Figure S1) exhibit more pronounced secondary modes than in the other regions that were reported. Here we speculate about two possible factors that contribute to the appearance of such double peaks in certain cross-sections. One is that our reasoning of optimized information transfer might be restricted only to individual fascicles or a limited subset of fascicles connecting functionally related regions. In such a case the cross sections of the nerves or white matter structures that include functionally unrelated fascicles, providing quite distinct connections, will be optimized independently and hence produce different distributions. Another possibility is that such multiple modes are the result of having the same cross section that contains both non-myelinated and myelinated axons. To account for the information transfer in those situations a more complex model needs to be developed, perhaps a simple extension, containing multiple unimodal distribution as described here. Use of multimodal distributions containing multiple non-skewed, e.g., normal distributions, cannot successfully explain the appearance of the experimentally observed ADDs. Also, the practical value of any multi-modal ADD models will be limited due to the presence of too many parameters and the overfitting that would be problematic when applied to noisy MRI data.

The above described optimum principle predicts a new family of skewed, heavy-tailed distributions that fit the experimental ADD data well, and better in terms of the overall least-squares deviation than the GD, LND, and PMD distributions. This finding extends also when the AIC or BIC are used. However, the TRD distribution, although better than IUBD in terms of LSE, failed to fit properly in an overwhelming number of attempts, when the initial conditions were varied. Since we are also looking for a robust distribution to be used in practical applications, such as AxCaliber MRI, our model of choice is still the IUBD.

Besides targeting different anatomical areas for detailed analysis of ADDs across the brain and generally across the central nervous system, an important area of future research is to see whether these skewed and heterogeneous distributions also manifest themselves during normal development. Moreover, is the deviation from IUBD or TD an indicator of abnormal development, and/or does it have diagnostic value as a marker of disease or degeneration? Do the coefficients that parameterize this new distribution have any biological significance? We believe that they might. We know that the ADD in the optic tract has a small variance, presumably to allow signals to be transmitted to the visual area at about the same time, whereas motor fibers can have a broad, skewed, and heavy-tailed appearance, to allow for large fibers to control rapid gross movements and smaller diameter fibers to control slower, finer movements. We would also like to apply this distribution framework to begin testing whether there are significant differences among ADDs in the PNS and CNS. An unresolved question we plan to investigate further is how maximum firing rate scales with axon diameter. While we assume a power-law relationship here, there is little data to confirm what the form of this relationship should be. Both theoretical models describing action potential propagation in myelinated axons and direct measurements could be used to address this important question.

The derived IUBD distribution to our knowledge is a novel one. Besides its biological relevance, it has many desirable practical and theoretical properties. Understanding the basis of its power to fit skewed functions of many forms and its robust convergence properties, i.e., its insensitivity to initial conditions, is important to understand. Incorporating this new distribution in our AxCaliber MRI framework is clearly an important next step and a detailed study addressing the applicability of the IUBD to AxCaliber MRI is currently underway and will be published independently. In this work, however, we want to emphasize more the biological and neuroscientific relevance of the information transfer optimization as a guiding principle in determining the shape of the ADDs in nerve fascicles.

## Supporting Information

Table S1 **The values of parameters obtained for the best fits found for each model and each data set.**
(PDF)

Figure S1 **Figure shows all ADDs in the EMD1 data set together with the best fits obtained for each of the models.**
(PDF)

Figure S2 **Figure shows all ADDs in the EMD2 data set together with the best fits obtained for each of the models.**
(PDF)

Figure S3 **Figure shows all ADDs in the EMD3 data set together with the best fits obtained for each of the models.**
(PDF)

Text S1 **A short description and a discussion of the results shown in Figures S1, S2, S3.**
(PDF)
