# Correction: An Optimum Principle Predicts the Distribution of Axon Diameters in Normal White Matter

**DOI:** 10.1371/annotation/295b3621-1d29-43ac-8180-6fad3d2de593

**Published:** 2013-12-17

**Authors:** Sinisa Pajevic, Peter J. Basser

There is an error in Equation 18 which appears under the sub-heading “Log-normal Distribution” in the “Introduction” section. The correct Equation 18 is:


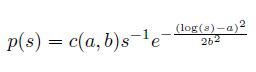


Additionally, there is an error in Table S1. Please view the correct Table S1 here 
