# Supplementary material for: An Optimum Principle Predicts the Distribution of Axon Diameters in Normal White Matter
Source: PLoS One. 2013 Jan 28;8(1):e54095. doi: 10.1371/journal.pone.0054095 (PMC3557303; doi:10.1371/journal.pone.0054095)
Supplement: Table S1 — The values of parameters obtained for the best fits found for each model and each data set. (PDF) [file pone.0054095.s001.pdf]

Dataset (#)	IUBD		TRD		GD		LND		PMD	
EMD1 (1)	a=3.6	b=7	a=11	b=0.067	a=-0.33	b=0.098	a=3.3	b=7.7	a=2.4	b=-11
EMD1 (2)	a=3.6	b=6.7	a=11	b=0.069	a=-0.3	b=0.099	a=3.3	b=7.4	a=2.2	b=-11
EMD1 (3)	a=2.3	b=3.9	a=7	b=0.11	a=-0.27	b=0.16	a=1.9	b=4.6	a=2.4	b=-6.7
EMD1 (4)	a=2.9	b=5.5	a=8.9	b=0.083	a=-0.32	b=0.12	a=2.6	b=6.3	a=2.4	b=-8.8
EMD1 (5)	a=3.7	b=7.6	a=12	b=0.062	a=-0.35	b=0.092	a=3.4	b=8.3	a=2.4	b=-11
EMD1 (6)	a=1.9	b=0.9	a=3.7	b=0.42	a=0.39	b=0.34	a=1.4	b=1.3	a=0.92	b=-3.4
EMD1 (7)	a=1.8	b=1.9	a=4.9	b=0.2	a=-0.046	b=0.25	a=1.4	b=2.6	a=1.8	b=-4.4
EMD1 (8)	a=1.6	b=1.8	a=4.6	b=0.22	a=-0.046	b=0.27	a=1.3	b=2.4	a=1.9	b=-4.2
EMD1 (9)	a=1.8	b=1.6	a=4.4	b=0.25	a=0.036	b=0.27	a=1.4	b=2.2	a=1.6	b=-4.2
EMD1 (10)	a=1.4	b=1.5	a=3.9	b=0.26	a=-0.04	b=0.32	a=1	b=2.1	a=2.1	b=-3.6
EMD1 (11)	a=2.5	b=3.3	a=6.9	b=0.13	a=-0.14	b=0.16	a=2.2	b=4	a=1.8	b=-6.6
EMD1 (12)	a=2.6	b=3.7	a=7.2	b=0.12	a=-0.19	b=0.16	a=2.2	b=4.4	a=2	b=-6.9
EMD1 (13)	a=1.9	b=2.6	a=5.4	b=0.16	a=-0.17	b=0.21	a=1.6	b=3.3	a=2.1	b=-5.3
EMD1 (14)	a=2.1	b=2.8	a=5.9	b=0.15	a=-0.14	b=0.19	a=1.8	b=3.5	a=2	b=-5.7
EMD1 (15)	a=3.2	b=5	a=9.1	b=0.091	a=-0.22	b=0.12	a=2.9	b=5.6	a=2	b=-8.8
EMD1 (16)	a=1.9	b=1.5	a=4.3	b=0.27	a=0.11	b=0.28	a=1.4	b=2	a=1.4	b=-4.1
EMD1 (17)	a=2	b=1.7	a=4.6	b=0.24	a=0.069	b=0.25	a=1.5	b=2.2	a=1.5	b=-4.4
EMD1 (18)	a=1.7	b=2.1	a=5	b=0.18	a=-0.12	b=0.24	a=1.4	b=2.8	a=2.1	b=-4.6
EMD1 (19)	a=1.7	b=1.8	a=4.7	b=0.22	a=-0.013	b=0.26	a=1.4	b=2.4	a=1.7	b=-4.3
EMD1 (20)	a=1.7	b=1.8	a=4.4	b=0.24	a=-0.011	b=0.27	a=1.3	b=2.3	a=1.8	b=-4.3
EMD1 (21)	a=2.5	b=3.2	a=6.7	b=0.14	a=-0.12	b=0.17	a=2.1	b=3.8	a=1.8	b=-6.4
EMD1 (22)	a=2	b=2.2	a=5.2	b=0.19	a=-0.055	b=0.22	a=1.6	b=2.8	a=1.8	b=-4.9
EMD1 (23)	a=1.7	b=2.5	a=5.3	b=0.16	a=-0.21	b=0.22	a=1.4	b=3.3	a=2.4	b=-4.9
EMD1 (24)	a=1.8	b=2	a=4.8	b=0.2	a=-0.06	b=0.25	a=1.4	b=2.6	a=1.9	b=-4.5
EMD1 (25)	a=2.1	b=2.6	a=5.7	b=0.16	a=-0.12	b=0.2	a=1.7	b=3.2	a=1.9	b=-5.4
EMD1 (26)	a=1.7	b=1.4	a=4	b=0.3	a=0.11	b=0.3	a=1.3	b=1.9	a=1.5	b=-3.8
EMD1 (27)	a=2.2	b=1.7	a=4.7	b=0.25	a=0.13	b=0.25	a=1.7	b=2.2	a=1.3	b=-4.6
EMD1 (28)	a=1.6	b=1.5	a=4.1	b=0.27	a=0.018	b=0.29	a=1.2	b=2.1	a=1.8	b=-3.9
EMD1 (29)	a=1.4	b=0.86	a=3.1	b=0.43	a=0.22	b=0.41	a=0.92	b=1.3	a=1.5	b=-2.9
EMD1 (30)	a=2	b=1.4	a=4.3	b=0.28	a=0.15	b=0.27	a=1.5	b=1.9	a=1.3	b=-4.2
EMD1 (31)	a=0.88	b=0.19	a=1.8	b=1.3	a=0.67	b=0.85	a=0.42	b=0.56	a=1.5	b=-1.6
EMD1 (32)	a=1.5	b=1.3	a=3.9	b=0.28	a=0.044	b=0.32	a=1.1	b=1.9	a=1.8	b=-3.6
EMD1 (33)	a=2.1	b=2.1	a=5.5	b=0.19	a=-0.00092	b=0.22	a=1.8	b=2.8	a=1.6	b=-4.9
EMD1 (34)	a=1.2	b=1.3	a=3.7	b=0.27	a=-0.057	b=0.35	a=0.88	b=2	a=2.3	b=-3.3
EMD1 (35)	a=2	b=2	a=5.4	b=0.19	a=-0.021	b=0.23	a=1.7	b=2.7	a=1.7	b=-4.8
EMD2 (1)	a=3.2	b=5	a=9	b=0.091	a=-0.23	b=0.12	a=2.8	b=5.7	a=2	b=-8.7
EMD2 (2)	a=3.8	b=4	a=8.8	b=0.11	a=-0.025	b=0.12	a=3.4	b=4.5	a=1.3	b=-8.6
EMD2 (3)	a=4	b=2.7	a=7.6	b=0.16	a=0.19	b=0.14	a=3.5	b=3.2	a=0.91	b=-7.4
EMD2 (4)	a=2.4	b=1.4	a=4.7	b=0.29	a=0.26	b=0.25	a=1.9	b=1.9	a=0.98	b=-4.5
EMD2 (5)	a=3.9	b=4.8	a=9.6	b=0.096	a=-0.1	b=0.11	a=3.5	b=5.4	a=1.5	b=-9.4
EMD2 (6)	a=4	b=4.2	a=9.3	b=0.11	a=-0.015	b=0.12	a=3.6	b=4.7	a=1.3	b=-9
EMD3 (1)	a=2.5	b=3.4	a=6.6	b=0.13	a=-0.15	b=0.16	a=2.1	b=4	a=1.9	b=-6.5
EMD3 (2)	a=2.2	b=3.2	a=6.4	b=0.14	a=-0.18	b=0.18	a=1.9	b=3.8	a=2	b=-6.1
EMD3 (3)	a=2.4	b=2.6	a=6.2	b=0.16	a=-0.036	b=0.19	a=2	b=3.2	a=1.6	b=-5.8
EMD3 (4)	a=2.4	b=1.8	a=5	b=0.24	a=0.15	b=0.23	a=1.9	b=2.3	a=1.2	b=-4.9
EMD3 (5)	a=3.2	b=2.4	a=6.6	b=0.18	a=0.13	b=0.17	a=2.7	b=2.9	a=1.1	b=-6.2

TABLE ST1: The values of parameters obtained for the best fits found for each model and each data set
