# Supplementary material for: An Optimum Principle Predicts the Distribution of Axon Diameters in Normal White Matter
Source: PLoS One. 2013 Jan 28;8(1):e54095. doi: 10.1371/journal.pone.0054095 (PMC3557303; doi:10.1371/journal.pone.0054095)
Supplement: Figure S1 — Figure shows all ADDs in the EMD1 data set together with the best fits obtained for each of the models. (PDF) [file pone.0054095.s002.pdf]

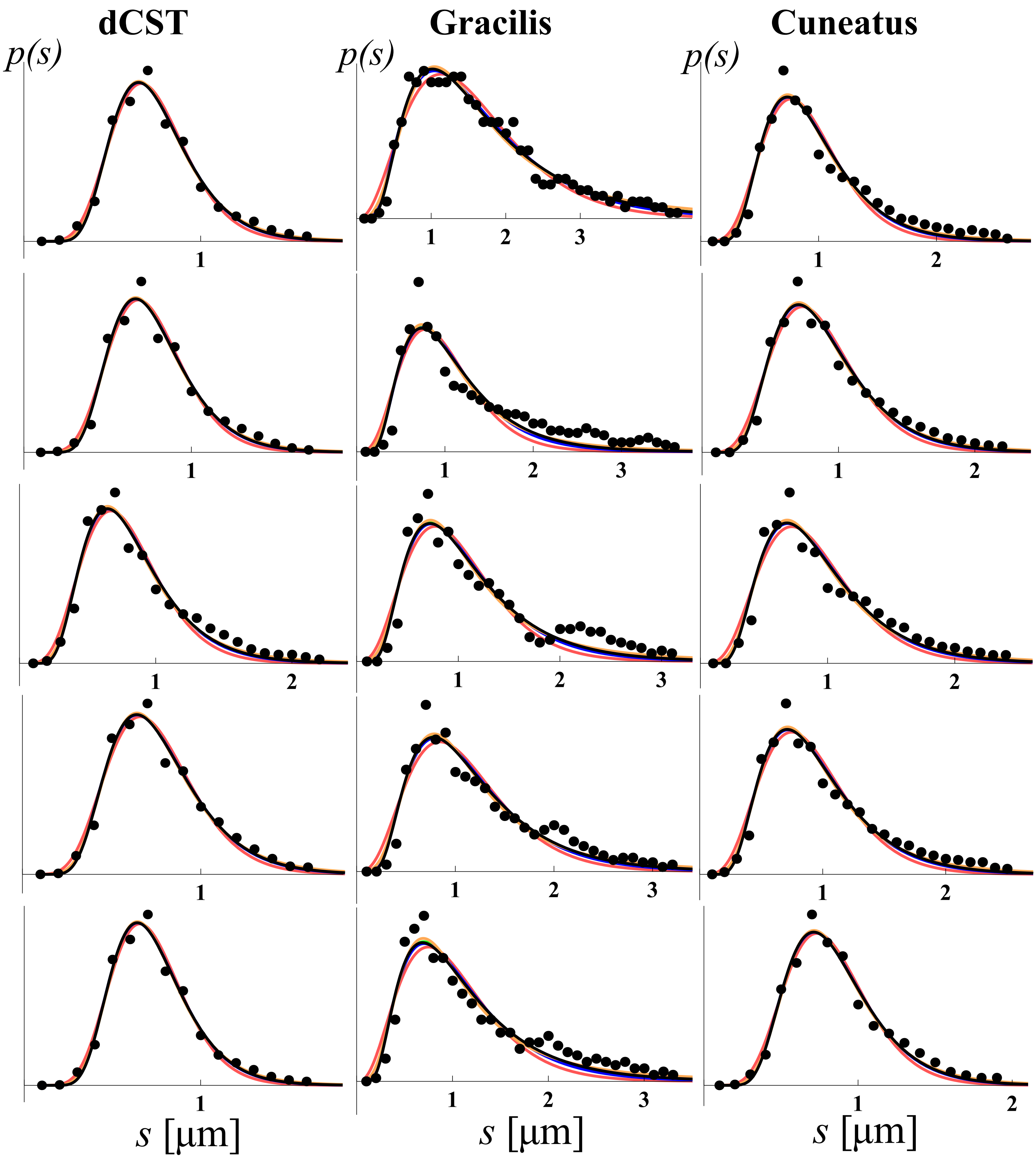

FIG. SF1: (Panel 1) Fits obtained for EMD1 (solid black circles) together with the fitted models displayed as follows: IUBD (black), TRD (orange), GD (red), LND (green), PMD (blue). The data contained ADDs for 7 anatomical white-matter regions and each of the regions had 5 replicates, hence in all there were 35 ADDs. To display the fits better this figure is split into two panels. The first panel displays 5 replicates in each of the three columns, which correspond to the different anatomical regions, dorsal corticospinal (dCST), gracilis (FG), and cuneatus (FC), as indicated. The remaining 4 areas are displayed on the next page.

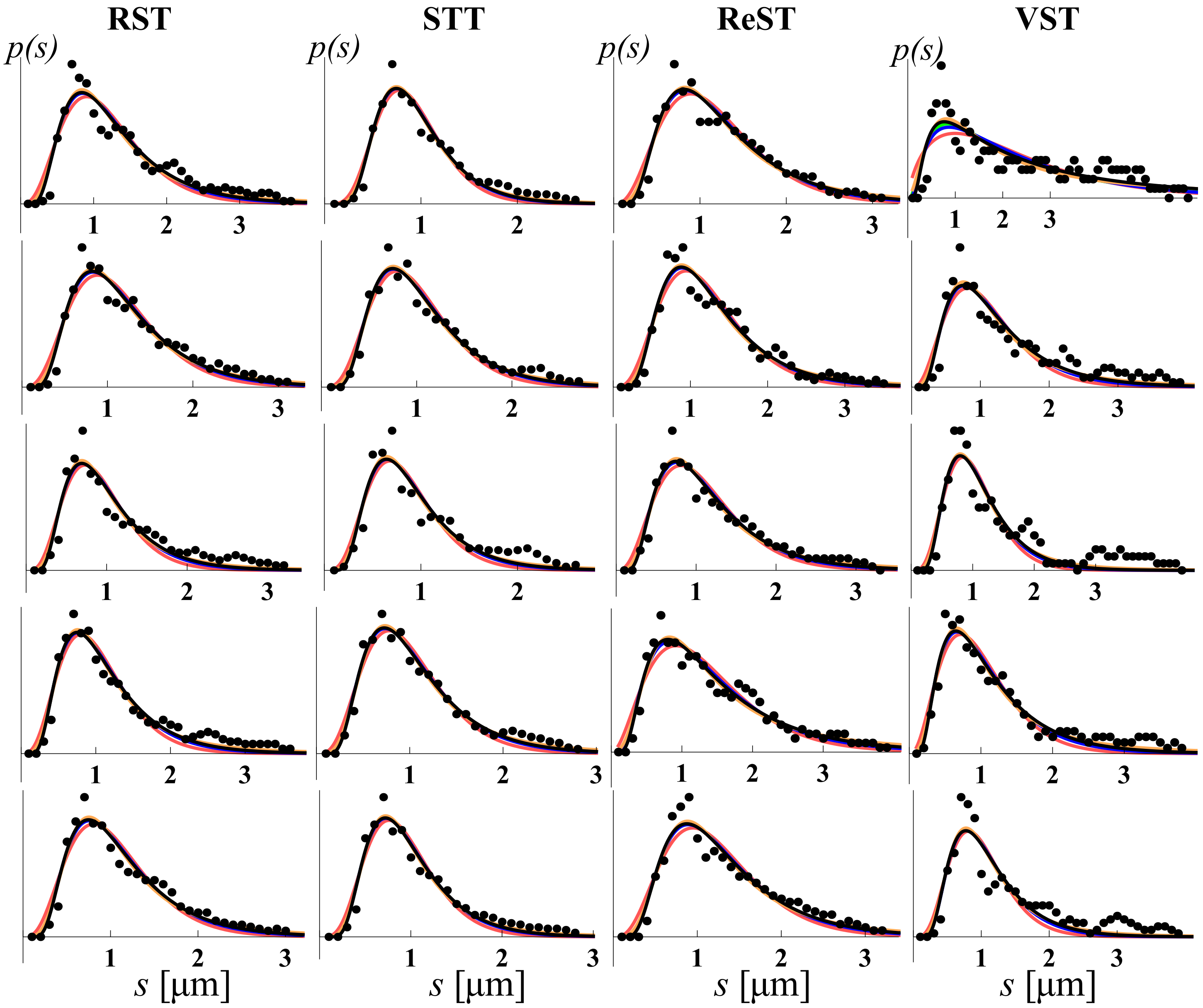

FIG. SF1: (Panel 2) Continuation of figure SF1 showing fits obtained for EMD1 (solid black circles) together with the fitted models displayed as follows: IUBD (black), TRD (orange), GD (red), LND (green), PMD (blue). The four columns displayed in this second panel correspond to four different anatomical regions: rubrospinal (RST), spinothalamic (STT), reticulospinal (ReST), and vestibulospinal (VST), as indicated.
