# Supplementary material for: An Optimum Principle Predicts the Distribution of Axon Diameters in Normal White Matter
Source: PLoS One. 2013 Jan 28;8(1):e54095. doi: 10.1371/journal.pone.0054095 (PMC3557303; doi:10.1371/journal.pone.0054095)
Supplement: Figure S2 — Figure shows all ADDs in the EMD2 data set together with the best fits obtained for each of the models. (PDF) [file pone.0054095.s003.pdf]

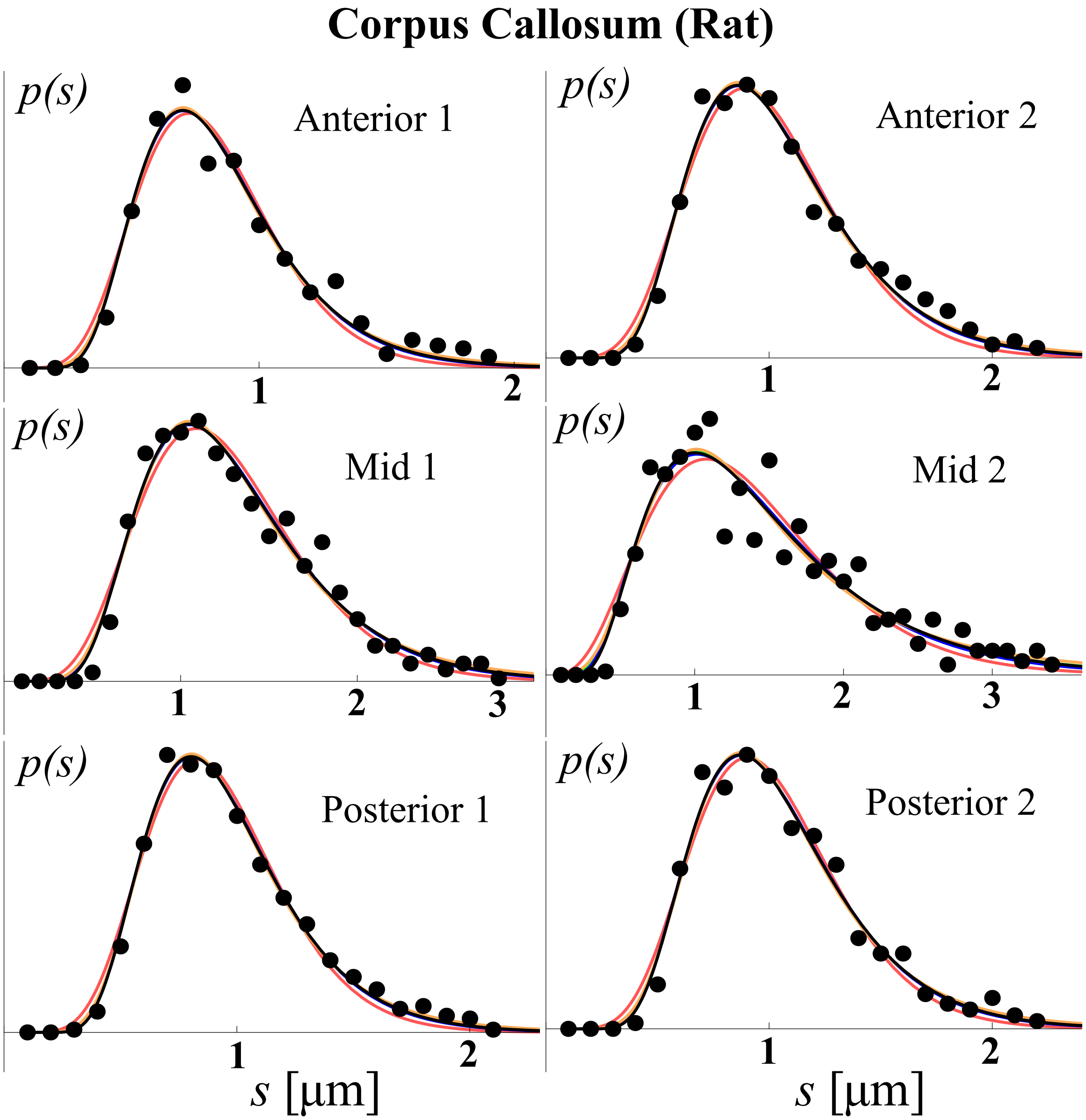

FIG. SF2: Best fits obtained for EMD2 for all ADDs and all models. The ADDs are from 6 consecutive subsections of the Corpus Callosum, ranging from anterior to posterior regions of corpus callosum (genu to splenium), so that the top row are anterior regions and the bottom row are posterior regions. The fitted models are displayed as follows: IUBD (black), TRD (orange), GD (red), LND (green), PMD (blue).
