# Supplementary material for: An Optimum Principle Predicts the Distribution of Axon Diameters in Normal White Matter
Source: PLoS One. 2013 Jan 28;8(1):e54095. doi: 10.1371/journal.pone.0054095 (PMC3557303; doi:10.1371/journal.pone.0054095)
Supplement: Figure S3 — Figure shows all ADDs in the EMD3 data set together with the best fits obtained for each of the models. (PDF) [file pone.0054095.s004.pdf]

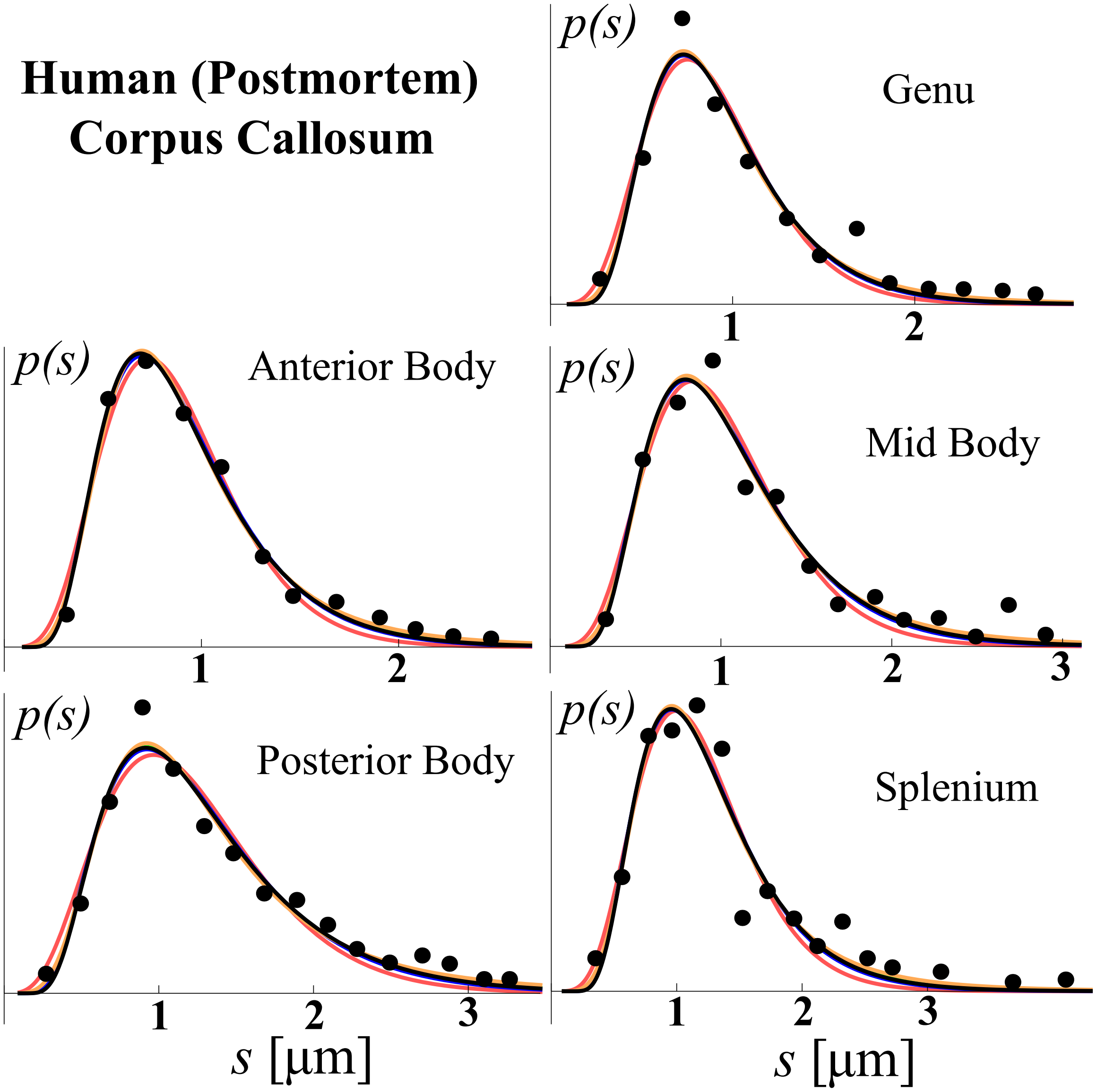

FIG. SF3: Best fits obtained for EMD3 for all ADDs and all models. The ADD curves ranging from anterior to posterior areas of corpus callosum are ordered from top to bottom, from Genu, Anterior Body, Mid Body, Posterior Body, to Splenium. The fitted models are displayed as follows: IUBD (black), TRD (orange), GD (red), LND (green), PMD (blue).
