# Supplementary material for: An Optimum Principle Predicts the Distribution of Axon Diameters in Normal White Matter
Source: PLoS One. 2013 Jan 28;8(1):e54095. doi: 10.1371/journal.pone.0054095 (PMC3557303; doi:10.1371/journal.pone.0054095)
Supplement: Text S1 — A short description and a discussion of the results shown in Figures S1, S2, S3. (PDF) [file pone.0054095.s005.pdf]

Supplementary Information: An optimum principle predicts the distribution of axon diameters in normal white matter

Sinisa Pajevic[1†] and Peter J. Basser[2‡]
[1†] *MSCL, DCB, CIT, NIH;* [2‡] *STBB, PPITS, NICHD, NIH*

In this supplementary material we provide a complete set of the fitting results, so that each individual data set can be assessed for the quality and quality of the fits. Prior to showing these fits we also provide a supplementary Table ST1, which displays the values of the estimated model parameter found after an extensive search. These were used as initial values for parameters when fitting the jackknife randomizations, and as the "optimal initial values" for the randomized initializations (the value of P_o in equation 20). As already emphasized in the main text, visual inspection is insufficient to discriminate between most of the models, except in the case of the gamma distribution which does not appear to be a good choice for the parametric form of ADDs.

Best fits obtained for all data sets and models

A total of 46 ADD curves, which are pooled from three different data sets as described in the main text (EMD1, 35 curves, shown in the two panels of figure SF1, EMD2, 6 curves shown in the figure SF2, and EMD3, 5 curves shown in the Figure SF3), were used to perform model fitting and comparison. For each curve and for each model we were able to attain a relatively good fit to the data after an extensive search. The significant deviations from the model appear in two forms, presence of outliers, as electron micrography (EM) cross sections often tend to have a few very large axons that in essence cannot conform to any unimodal analytical parametric model. Note that the curves presented here have already been truncated once the ADD appeared to have significantly decayed away from its peak. Few of the data sets, however, appear genuinely multimodal, they are mostly dependent on the type of nerve/anatomical section in which ADD is measured. For example, the ADDs measured in gracilis (in Panel 1 of Figure SF1) and vestibulospinal tract (VST) (in Panel 2 of Figure SF1) exhibit more pronounced secondary modes than in the other regions that were reported. In the main text it has been discussed that this could be the result of the presence of very distinct fascicles in a given cross-section of a nerve, as well as a possibility that the cross-section contains both non-myelinated and myelinated axons. Additionally, several replicates in gracilis do not conform well to any of the models studied here as they show very pronounced peaks in the distribution that none of the models could accommodate. Thus it is possible that the ADDs in nerve fascicles in different anatomical positions are guided and organized by different principles. Studying this further would require acquisition of many EM replicates from the same anatomical position, and performing the same model comparison presented here.

Randomization comparisons for each ADD using the jackknife

As described in the main text, we use the jackknife (JK) to make statistical comparisons (one-sided t-tests) for each individual ADD. Since the complete set of ADDs is provided here, we supplement the results in Figure 2c by identifying specific ADDs in which IUBD failed to be declared significantly better. When referring to replicates in the EMD1 data set, for each anatomical region, the replicate number indicates the row number, hence the replicate 1 is in the top row. IUBD failed to be declared significantly better than LND for 16 out 46 ADDs. Of those 16, 12 ADDs were in EMD1 and 4 in EMD3. More specifically, in EMD1 the following ADDs failed to be declared significantly better: all five replicates in dCST, the replicates 1 and 3 in STT, and the replicate number 3 in VST. In EMD3 all replicates failed to be declared significantly better, except for the second one (anterior body). Note that ADD in dCST had much smaller fibers and less skewed distribution than other regions. When the comparison is made between IUBD and PMD, IUBD failed to be declared significantly better in 5 out of 46 ADDs. Specifically, in EMD1 the ADD replicates 1, 2, and 5 from dCST, and in EMD3 the ADDs from the genu and the splenium (the most anterior and the most posterior).

Supporting Information Legends

TABLE ST1: The values of parameters obtained for the best fits found for each model and each data set

FIG. SF1: Model fits for all ADD curves in EMD1 data set.

FIG. SF2: Model fits for all ADD curves in EMD2 data set.

FIG. SF3: Model fits for all ADD curves in EMD3 data set.
